# Synthesis of High Performance Thiophene–Aromatic Polyesters from Bio-Sourced Organic Acids and Polysaccharide-Derived Diol: Characterization and Degradability Studies

**DOI:** 10.3390/molecules27010325

**Published:** 2022-01-05

**Authors:** Lesly Dasilva Wandji Djouonkep, Arnaud Kamdem Tamo, Ingo Doench, Naomie Beolle Songwe Selabi, Emmanuel Monga Ilunga, Arnaud Regis Kamgue Lenwoue, Mario Gauthier, Zhengzai Cheng, Anayancy Osorio-Madrazo

**Affiliations:** 1 Institute of Fine Organic Chemistry and New Organic Materials, Wuhan University of Science and Technology, Wuhan 430081, China; dasilvasilva3008@wust.edu.cn (L.D.W.D.); gauthier@uwaterloo.ca (M.G.); 2 Department of Petroleum Engineering, Applied Chemistry in Oil and Gas Fields, Yangtze University, Wuhan 430100, China; 3 Laboratory for Bioinspired Materials—BMBT, Institute of Microsystems Engineering—IMTEK, University of Freiburg, 79110 Freiburg, Germany; arnaud.kamdem@imtek.uni-freiburg.de (A.K.T.); ingo.doench@imtek.uni-freiburg.de (I.D.); 4 Freiburg Materials Research Center—FMF, University of Freiburg, 79104 Freiburg, Germany; 5 Freiburg Center for Interactive Materials and Bioinspired Technologies—FIT, University of Freiburg, 79110 Freiburg, Germany; 6 Institute of Advanced Materials and Nanotechnology, Wuhan University of Science and Technology, Wuhan 430081, China; naobelle123@wust.edu.cn (N.B.S.S.); emmanuelmonga64@gmail.com (E.M.I.); 7 National Engineering Laboratory of Petroleum Drilling Technology, Department of Petroleum Engineering, Leak Resistance & Sealing Technology Research Department, Yangtze University, Wuhan 430100, China; regiskamgue@yahoo.fr; 8 Department of Chemistry, Institute for Polymer Research, University of Waterloo, 200 University Avenue West, Waterloo, ON N2L 3G1, Canada; 9 Coal Conversion and New Carbon Materials Hubei Key Laboratory, Wuhan University of Science and Technology, Wuhan 430081, China

**Keywords:** bio-based polyesters, (thiophene–aromatic) copolyesters, diester monomers, dimethyl 2,5-thiophenedicarboxylate (DMTD), dimethyl 2,5-dimethoxyterephthalate (DMDMT), bio-based 1,6-hexanediol, thermal/mechanical properties, polyester (bio)degradation

## Abstract

In this work, the feasibility of replacing petroleum-based poly(ethylene terephthalate) (PET) with fully bio-based copolyesters derived from dimethyl 2,5-thiophenedicarboxylate (DMTD), dimethyl 2,5-dimethoxyterephthalate (DMDMT), and polysaccharide-derived 1,6-hexanediol (HDO) was investigated. A systematic study of structure-property relationship revealed that the properties of these poly(thiophene–aromatic) copolyesters (PHS(20–90)) can be tailored by varying the ratio of diester monomers in the reaction, whereby an increase in DMTD content noticeably shortened the reaction time in the transesterification step due to its higher reactivity as compared with DMDMT. The copolyesters had weight-average molar masses (Mw) between 27,500 and 38,800 g/mol, and dispersity Đ of 2.0–2.5. The different polarity and stability of heterocyclic DMTD provided an efficient mean to tailor the crystallization ability of the copolyesters, which in turn affected the thermal and mechanical performance. The glass transition temperature (T_g_) could be tuned from 70–100 °C, while the tensile strength was in a range of 23–80 MPa. The obtained results confirmed that the co-monomers were successfully inserted into the copolyester chains. As compared with commercial poly(ethylene terephthalate), the copolyesters displayed not only enhanced susceptibility to hydrolysis, but also appreciable biodegradability by lipases, with weight losses of up to 16% by weight after 28 weeks of incubation.

## 1. Introduction

Commodity polymers are ubiquitous in our lives, and current state-of-the-art on polymers include multiple studies devoted to the development of new approaches for the synthesis of man-made polymeric materials with precise structures and functions [1,2,3,4]. For example, fabrics derived from polyester fibers are widely used in clothing and furnishing, from shirts and pants to jackets and hats, sheets, blankets, upholstery, and computer mouse pads. Industrially, polyester ropes are also used to reinforce automotive tires, as conveyor belt fabrics, for seat belts, coated fabrics, and as high energy-absorbing plastic reinforcement. To meet current societal requirements, chemical and structural modifications are commonly made to polyesters to improve their properties. To this end, the modification of aromatic polyesters to make them more sustainable and biodegradable is a challenging task that has been extensively pursued in recent years [5,6]. Aromatic polyesters like poly(ethylene terephthalate) (PET) [7] or poly(butylene terephthalate) (PBT) [8] are widely recounted as premium thermoplastic materials with excellent thermal, mechanical, and optical properties among others, but their degradation in natural environments is insignificant. While these high-performance materials have enabled transformative progress, most of them still involve petroleum-based chemicals as monomers, which have antagonistic impacts on the environment.

Recently, by taking advantage of fundamental differences in alternative renewable assets (e.g., sulfur as a by-product of the oil and gas industry, and different biomass derivatives), research has turned to the design of novel economical thiophene–aromatic polymers, thereby converting the byproduct sulfur to renewable raw materials [9,10]. Many compounds with a low cost, environmental friendliness, diverse chemical structures, and many reactive functional sites, derived from biomass with broad availability, have been used in the synthesis of new bio-based polyesters [11,12]. In this context, biomass byproducts have proven to be valuable resources to replace fossil fuels effectively. The substitution of fossil-based resources with molecules extracted from the biomass represents one of the most important targets of green chemistry [13,14,15,16,17]. This replacement is associated with two main goals: the use of renewable sources instead of fossil ones, and the development of a low-carbon economy geared towards reducing waste and minimizing resources consumption [18,19,20,21,22,23]. Nevertheless, contemporary requirements necessitate further research to introduce additional chemical functionalities exhibiting a higher reactivity as compared with currently available, petroleum-based sources [24,25,26,27,28]. Consequently, the opportunity to create bio-based polymers to replace non-renewable polyesters, by partially replacing diols or diacids, or even both, with bio-based monomers is extremely attractive, as long as the main properties of these unique polymers are not significantly impacted [29].

Thiophene–aromatic polymers are a class of conjugated polymers including thiophene-based units and aromatic units along their backbone. The thiophene rings often include alkyl substituents as solubilizing moieties, to improve the processability of the polymers [30,31]. The selection of thiophene as a co-monomer in conjugated polymers can be justified by different factors. It is readily available, and the chemistry of thiophene is well-developed and understood, allowing its functionalization in a wide range of reactions [32]. Similarly, copolymerization is a well-understood and efficient method to increase the bio-based carbon content of materials. Many renewable bio-based monomers, such as lactic acid [33,34], 2,5-furandicarboxylic acid (FDCA) [35] and 2,5-thiophenedicarboxylic acid (TDCA) [36] have been developed to face the depletion of oil supplies. In contrast, very few investigations have concerned thiophene–aromatic copolyesters, such as poly(propylene naphthalate-*co*-propylene 2,5-thiophenedicarboxylate) [37], poly(butylene 2,5-thiophenedicarboxylate-*co*-1,4-cyclohexanedimethylene 2,5-thiophenedicarboxylate) [38], poly(trimethylene 2,5-thiophenedicarboxylate-*co*-trimethylene terephthalate) [39], and poly(*p*-phenylene 3,4-disubstituted-2,5-thiophendicarboxylate) [40], which are reported to have good thermal stability and mechanical properties, and to display some degree of biodegradability in natural environments. Unfortunately, most of these polyesters exhibit low processing temperatures (<200 °C), low molar masses and moduli, and in most cases show no crystallization due to the rigid units breaking the regularity of the chains [41]. As compared with their FDCA-based analogs, polyesters derived from TDCA display enhanced crystallization ability and ductility [42]. These superior properties were attributed to the lower dipole moment of the less electronegative S atom relative to O (0.51 D vs. 0.70 D), differences in electron mobility, and higher average electronic coupling for thiophene-based as compared with furan-based polyesters.

2,5-Thiophenedicarboxylic acid (TDCA) is a stable thiophene derivative which can be synthesized from polysaccharides, a renewable resource. However, the TDCA currently sold on the market is generally synthesized from adipic acid and thionyl chloride [43]. TDCA and terephthalic acid are very similar, in that their chemical structures contain cyclic conjugated systems. In this investigation, TDCA was converted to dimethyl 2,5-thiophenedicarboxylate (DMTD), a more reactive monomer, by esterification with methanol and sulfuric acid [44]. The resulting polymers contain five- and six-membered ring structures, which explains why these copolyesters have a performance similar to poly(ethylene terephthalate). The introduction of C-S polar bonds in the polyester backbone also improved their degradability. Furthermore, 1,4-cyclohexanedione-2,5-dicarboxylate is a derivative of bio-sourced succinic acid (SA), which can be converted easily to dimethyl 2,5-dimethoxy terephthalate (DMDMT) [45,46].

In our previous research [47], we investigated the synthesis and the characterization of a series of thiophene–aromatic copolyesters obtained with a 1:1 molar ratio of DMTD and DMDMT, while varying the diol chain length in the copolyester backbone. Despite their good thermal and mechanical properties, the degradability of the copolyesters under composting conditions was low. We further investigated the influence of composition on the properties of copolyesters derived from DMTD, DMDMT, and HDO, by varying the proportions of the diesters in the reaction. This approach was selected for two main reasons: the production of the bio-based 1,6-hexanediol (HDO) has been found to reduce greenhouse gas emissions and environmental impacts considerably; and since it can be derived from polysaccharides, the insertion of this diol could promote the biodegradability of copolyesters under the action of specific enzymes, such as Pseudomonas fluorescens. 1,6-Hexanediol (HDO) can be synthesized via hydrogenolytic ring opening of 5-hydroxymethylfurfural (HMF), a dehydration product of hexoses or reducing-end units of polysaccharides [48,49]. 5-Hydroxymethylfurfural (HMF) formation from carbohydrates has been the topic of several studies [50,51,52]. The degradation of biomass polysaccharide materials like cellulose [53,54], chitin [50] and chitosan [51,52,55,56], which yields reducing ends utilized in HMF synthesis was reported. The production of HMF from chitin biomass has been recently reviewed by Zhou et al. [50]. Besides, the depolymerization of chitosan by nitrous deamination can facilitate the cleavage of the glycosidic bond next to the produced 2,5-anhydro-D-mannofuranose (AMF) unit and, thereby the formation of HMF [51,52]. To this end, we now report the synthesis and the characterization of novel poly(hexamethylene 2,5-thiophenedicarboxylate-*co*-hexamethylene 2,5-dimethoxyterephthalate) copolyesters [PHS(20–90)], prepared by varying the molar ratio of thiophene units (20; 30; 50; 70; and 90%) incorporated in the copolyesters. The resulting thiophene–aromatic copolyesters exhibit properties comparable to commercial PET, as well as polypropylene (PP) and high-density polyethylene (HDPE), in addition to good biodegradability.

## 2. Results and Discussion

### 2.1. Synthesis of Copolyesters

**Figure 1:**
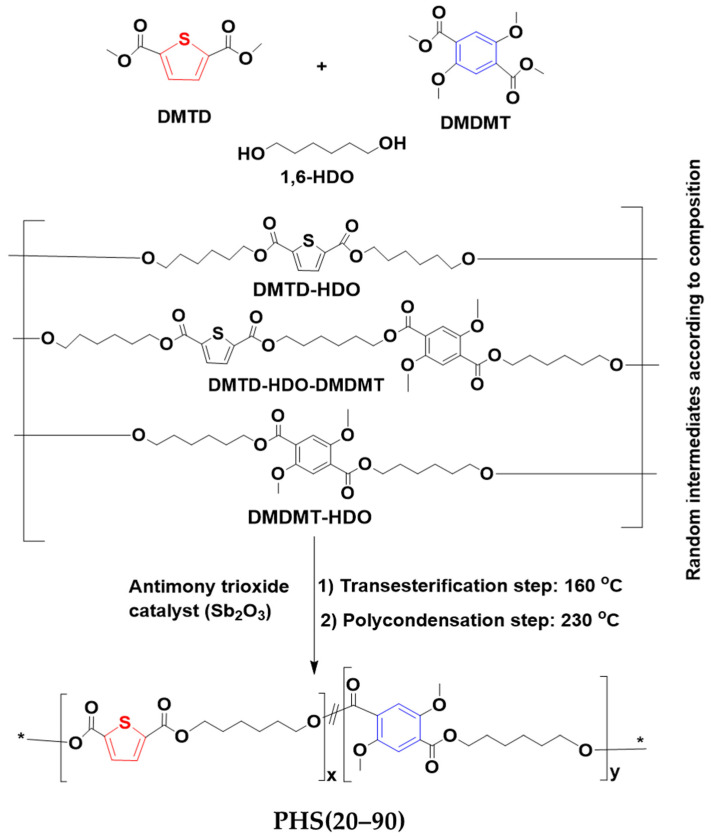
Synthesis of PHS(20–90) copolyesters.

**Figure 2:**
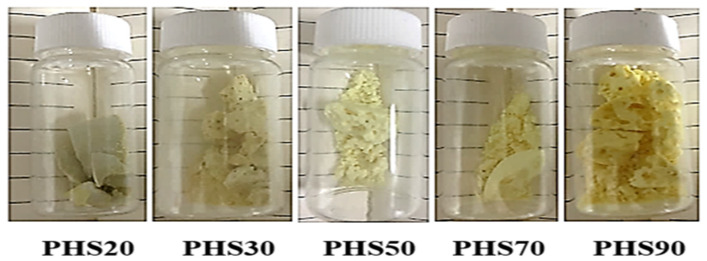
Appearance of obtained copolyesters PHS(20–90).

The synthetic route followed in this work for the preparation of the PHS(20–90) copolyesters, presented in Figure 1, is a two-step melt polycondensation of dimethyl 2,5-thiophenedicarboxylate (DMTD) and dimethyl 2,5-dimethoxyterephthalate (DMDMT) with 1,6-hexanediol (HDO), in the presence of antimony trioxide (Sb_2_O_3_) as catalyst. The molar ratio of thiophene (DMTD) was varied from 0.2 to 0.9, and the total feed ratio of diol:diester was set to 1.3:1, to ensure that the growing chains were terminated with hydroxyl groups throughout the polymerization. Since DMTD is unstable at high temperatures, it was necessary to limit the reaction temperature to 230 °C, to minimize thermal degradation. Nevertheless, as shown in Table 1, the polydispersity tends to increase, while an increase in DMTD amounts contributed to an increase in the reaction rate (time to reach the clearing point) at higher temperatures. This is potentially due to the higher reactivity of DMTD, based on the trends observed in the first two entries (both done at 160 °C) and the last two entries (both done at 180 °C). A different mobile phase (DMF) was selected for the GPC analysis of copolyesters PHS20 and PHS30, due to differences in solubility arising for higher DMDMT contents, to ensure reliable molar mass measurements. The PMMA-equivalent M_w_ of the copolyesters ranged from 27,500 to 38,800 g/mol, and the dispersity (Đ) was between 2.0 and 2.5. Sample PHS50 had the highest molar mass, which could be related to the equimolar amounts of DMTD and DMDMT as rigid backbone components, displaying maximum reactivity with the flexible diol spacer. The appearance of copolyester samples PHS(20–90) is compared in Figure 2. Their color varied from light brown to yellow for increasing DMTD contents.

### 2.2. Fourier Transform Infrared (FTIR) Spectroscopy

**Figure 3:**
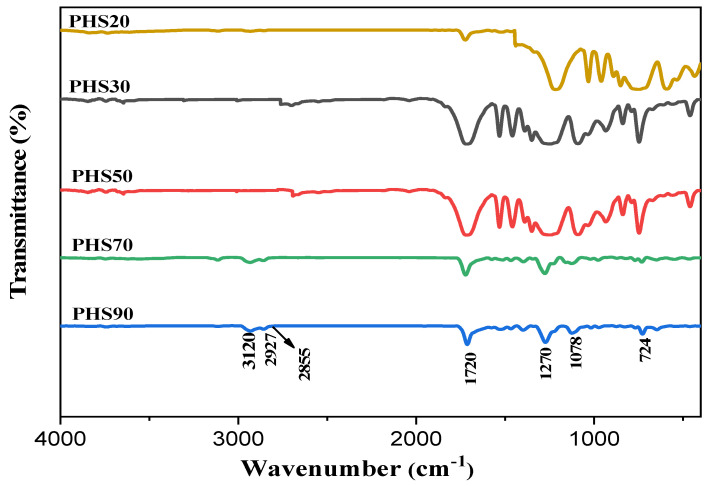
FTIR spectra for copolyesters PHS(20–90).

Infrared spectra of the copolyesters PHS(20–90), obtained by melt polymerization, are displayed in Figure 3. The spectra displayed various absorption peaks also characteristic for the diester precursors, as depicted in Figure 3 and summarized in Table 2. The =CH stretching peak for the thiophene ring appears at 3120 cm^−1^, while the -CH_2_- asymmetric and symmetric stretching peaks are observed at 2927 and 2855 cm^−1^, respectively. All the spectra display characteristic ester signals, including a strong C=O stretching absorption at 1720 cm^−1^ and two strong C-O absorption bands, around 1270 cm^−1^ for the sp^2^ stretch and at 1078 cm^−1^ for the sp^3^ stretch. The peaks at 1453 cm^−1^ and 1071 cm^−1^ correspond to the aromatic C=C stretching for DMDMT. A C-S absorption is also observed at 724 cm^−1^. It is obvious from the FTIR spectra that all the copolyesters include key absorption bands characteristic for both diester precursors, proving the successful incorporation of both monomers in the polyester backbone. It was also noticed that the bands characteristic for thiophene gradually become stronger with the DMTD content in the reactions, corresponding to increased DMTD incorporation.

### 2.3. ^1^H and ^13^C Nuclear Magnetic Resonance (NMR) Spectroscopy

**Figure 4:**
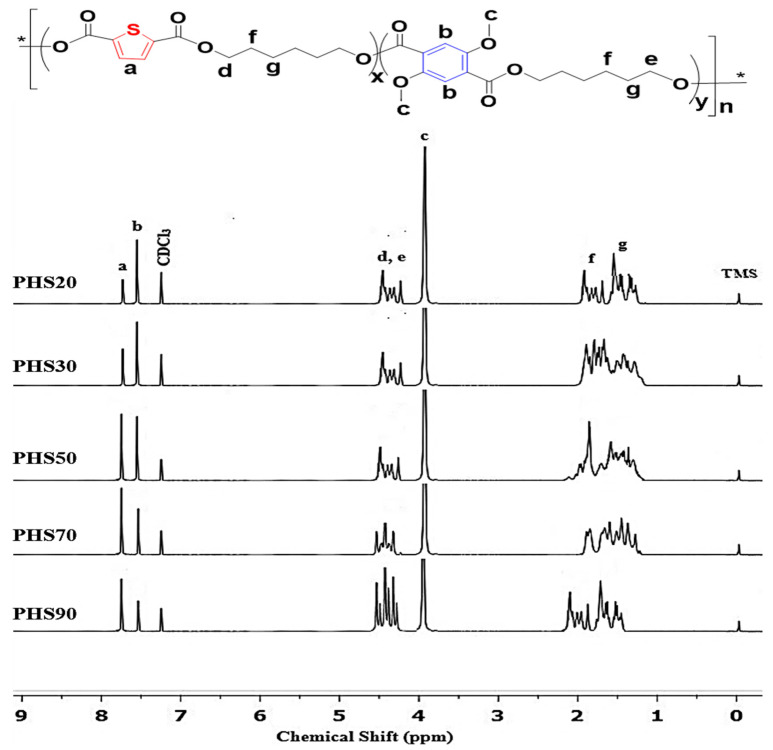
^1^H NMR spectra for copolyesters PHS(20–90). *: polymer chain extension.

**Figure 5:**
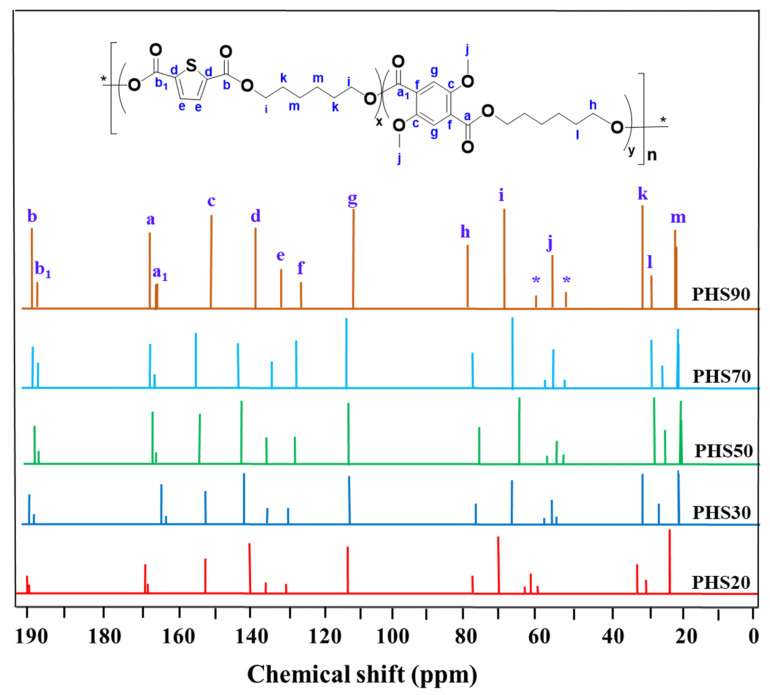
^13^C NMR spectrum for copolyester PHS(20–90). *: polymer end-chains signals.

The ^1^H NMR spectra and signal assignments for the different copolyesters synthesized are presented in Figure 4. The molar fractions of thiophenedicarboxylate and phenyl were calculated from the intensity ratios of baseline resolution signals H_a_ and H_b_ (Table 1), which were similar to those reported in the literature [57]. The average sequence length of the number of aromatic and aliphatic units could not be calculated based on the signal strength of O-CH_2_-protons (signal H_d_ and H_e_) for each structure due to the direct overlapping of signals when using 600 MHz instruments. Characteristic signals in the ^1^H NMR spectra are observed for -CH- protons on the thiophene ring (signal labelled as (a) at 7.77–7.80 ppm, while they are found at 7.55–7.62 ppm (b) for DMDMT. The -CH_2_- units connected to ester bonds produce characteristic signals at 4.30−4.43 ppm (d, e). The -OCH_3_ peak is located at 3.81–3.88 ppm (c), and -CH_2_- units disconnected from the ester bonds are detected at 1.79 and 1.45 ppm (f, g), respectively. The chloroform (CHCl_3_) peak is at 7.26 ppm, and the TMS reference signal is at 0 ppm. All the signals assigned in Figure 5 and listed in Table 3 again confirm the successful synthesis of the target products.

Analysis of the ^13^C NMR spectra likewise reveals various signals attributed to carbon atoms linked to different functional groups in the copolyester chains (Figure 5). Using the spectrum obtained for PHS90 as an example (Figure 5), these include peaks at 190.1 and 189.9 ppm (b, b1), corresponding to the quaternary carbonyl ester groups of thiophene; while 165.2 and 164.2 ppm (a, a1) belong to the carbonyl ester final groups (C=O ester) of phenyl group; 149.5, 134.0, 135.0, 143.1 ppm (d, e) correspond to aromatic thiophene carbons, 153.0, 127.6, 115.9 ppm (c, f, g) correspond to phenyl carbons, 55.5 ppm (j, O-CH_3_), 70.0, 64.8 ppm (i, h, -OCH_2_-), and the peaks at 31.7, 29.5, 29.0, 25.5 ppm (k, l, m) correspond to aliphatic carbons. The peaks at 62.3 and 58.1ppm (*) corresponds to the polymer end-chains. Unfortunately, due to the long (C_6_) diol spacers used, the ^13^C NMR spectra do not contain sufficient information to allow microstructure analysis of the copolymer chains.

### 2.4. Thermal Analysis

**Figure 6:**
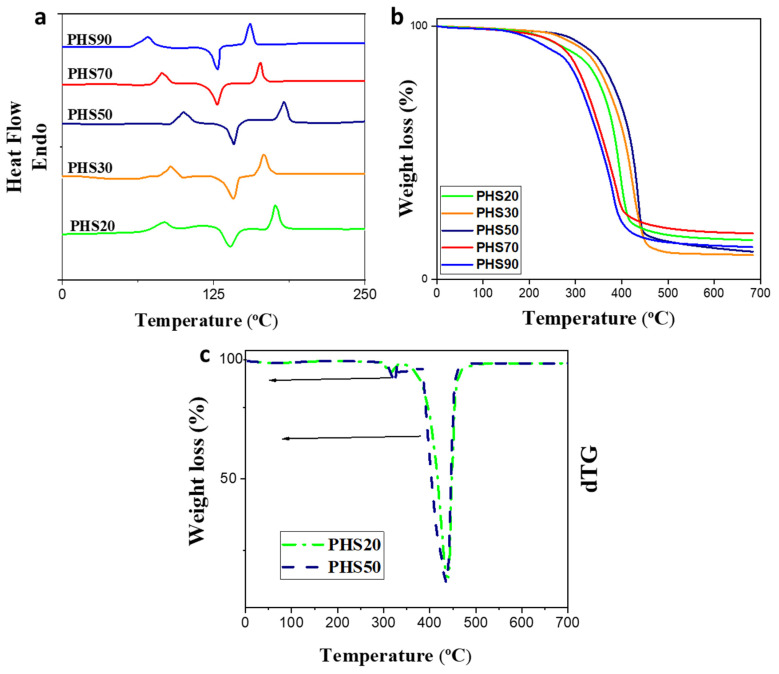
(**a**) DSC and (**b**) TGA curves for copolyesters PHS(20–90), (**c**) differential TGA curves for copolyesters PHS20 and PHS50.

DSC traces obtained for the copolyesters are provided in Figure 6a. The copolyesters were semi-crystalline, and the glass transition temperature (T_g_) varied between 70 and 100 °C, as determined by the diester composition and the presence of 1,6-hexanediol spacers controlling backbone flexibility. Annealing the copolyesters prior to the measurements did not afford reproducible results due to the influence of variations in their crystallinity level. The T_g_ of the copolyesters were more clearly detectable and more reproducible in the first DSC heating cycle when the samples were previously quenched from the melt, so as to be mainly amorphous in their initial state. A rise in T_g_ is observed for increasing DMTD contents up to PHS50, reaching a peak value of 100 °C, followed by drops for PHS70 and PHS90 (Figure 6a, Table 4). Rigid aromatic compounds, such as DMDMT, increase steric hindrance to chain segment rotation due to the benzene ring, which makes this compound suitable as a bio-based monomer for the preparation of polycondensates with high glass transition temperatures [58,59]. As compared with series of analogues derived from TDCA by Wang et al. [37,38], random poly(propylene naphthalate-*co*-propylene 2,5-thiophenedicarboxylate), poly(butylene 2,5-thiophenedicarboxylate-*co*-1,4-cyclohexanedimethylene 2,5-thiophenedicarboxylate), and poly(trimethylene 2,5-thiophenedicarboxylate-co-trimethylene terephthalate) polyesters were obtained with T_g_’s of 82.7, 69.2 and 47.3 °C, respectively, did not achieved higher T_g_ than PHS50, PHS30 and PHS 20 due to the rigid units breaking the regularity of the chains. This high T_g_ incurred herein is partly induced by the insertion of 1,6-hexanediol as flexible chain moieties which have proven to lower the transition temperature and strongly enhanced the crystalline nature of the polymers on the side-chain length.

All the copolymers were semi-crystalline in nature, with crystallization peaks of 126–142 °C and melting points of 155–183 °C (Figure 6a, Table 4). Similar to the trend observed in the T_g_ values, a maximum T_m_ (183 °C) was observed for PHS50, followed by drops for PHS70 and PHS90. The exothermic phase transition observed upon heating the samples above their T_g_ corresponds to cold crystallization, occurring once molecular mobility is sufficient to allow the formation of crystalline domains [60,61,62,63]. It has been suggested that an even number of carbon atoms in the diol (such as in 1,6-hexanediol) units may increase the crystallization rate of polyesters [64,65], which would facilitate the observation of the cold crystallization peaks (with corresponding enthalpy values ∆H_c_ summarized in Table 4) for all the samples. Since the areas of the crystallization and melting peaks were identical within 5% for each DSC trace, residual crystallinity was low or negligible after quenching. Under these conditions, the maxima observed in T_g_ for PHS50 are more likely the direct result of chain flexibility variations with the copolymer composition. The higher melting point of PHS50 may be associated with a higher degree of perfection of the crystalline phase due to enhanced intermolecular interactions. The melting point of PHS50 (183 °C) is comparable to poly(propylene naphthalate-*co*-propylene 2,5-thiophenedicarboxylate) (181 °C) reported by Guidotti et al. [64]. Consequently, the PHS50 copolymer, with a 1:1 molar ratio of each diester monomer, should likewise be of interest for industrial applications.

Relatively small variations in the decomposition temperatures (T_d, 5%_, T_d, 50%_, and T_d, max_) were observed for the different DMTD:DMDMT ratios (Figure 6b, Table 4), with a single-step decomposition process for all the samples (differential TGA curves provided for PHS20 and PHS50 in Figure 6c as examples) with only minor influences of the diesters molar ratio on the decomposition temperatures. The high thermal stability of the copolyesters is attributed to the presence of rigid phenyl and thiophene units in the copolymer chains [63], while the presence of DMDMT in the chains improved the thermal stability of the copolyesters to some extent, all the materials had relatively similar thermogravimetric behaviors, indicating that the aliphatic diol is likely the main component determining the thermal stability of the ester bonds, in agreement with the findings of Wang et al. [46]. Furthermore, the thermal stability of the PHS(20–90) copolymers is higher than for their 2,5-furandicarboxylic acid (FDCA) analogs reported by Knoop et al. [65]. The reason for the difference could lie in the larger bond angle between thiophene versus furan (148 °C as compared with 125 °C), and the higher electronegativity of O (0.70 D) relative to S (0.51 D), inducing a larger dipole moment in the furan ring than the thiophene ring.

Thermogravimetric analysis (Figure 6b,c and Table 4) yielded high decomposition temperatures for all the copolyesters (5% weight loss T_d, 5%_ ≥ 270 °C), which provides a very broad processing window for these materials. Furthermore, a 50% weight loss (T_d, 50%_) was only obtained in the 387–419 °C range, again demonstrating the good thermal stability of the copolymers.

### 2.5. Mechanical Analysis and Tensile Properties

**Figure 7:**
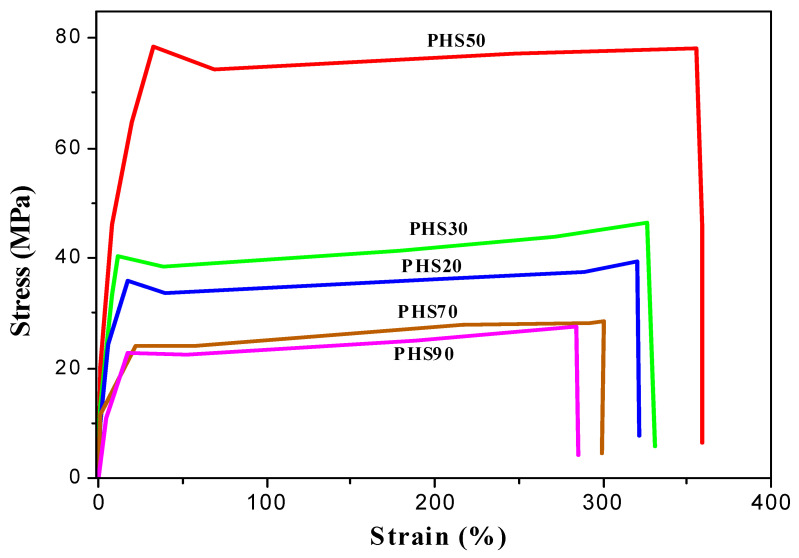
Tensile testing of synthesized copolyesters PHS(20–90).

The stress–strain curves obtained for copolyesters PHS(20–90) are provided in Figure 7. The corresponding extracted data, summarized in Table 5, show that PHS20 is the closest analogue of poly(ethylene terephthalate) (PET) among the synthesized polyester samples. The stress–stain curves of all the copolyesters display a clear yield point, but sample failure occurs before much stress hardening is observed. For this reason, relatively low elongation at break values were observed. On the basis of the data reported in Table 5, the tensile properties of polyester PHS50, a DMTD analogue of PET [66], are superior to that material. Not only does PHS50 have a higher T_g_, but it is also stiffer. The tensile strength and elongation at break values of the copolyesters progressively decrease as their DMTD content increases. While PHS50 has the highest yield stress among the copolyesters investigated, PHS70 and PHS90 have very similar behaviors and notably poorer tensile properties. These results are much better than that of poly(ethylene-*co*-2,2,4,4-tetramethyl-1,3-cyclobutanediol 2,5-furandicarboxylate) (PETF) synthesized by Wang et al. [37], exhibiting a tensile strength of only 69 MPa and an elongation at a break of 270%. The excellent performance of the present samples is due to the differences in crystallinity of the polymers containing thiophene and aromatic rings, and to the length of alkyl spacers. In making further comparisons of the PHS copolyesters with commercially available petroleum-based polymers (Table 5), we notice that high-density polyethylene (HDPE) and low-density polyethylene (LDPE) display similarities to some of the synthesized copolymers [66,67,68]. These results clearly show that copolyesters derived from bio-sourced monomers, such as DMTD and DMDMT, have great potential as replacement materials for commercial polymers derived solely from petrochemicals.

### 2.6. Degradation of Copolyesters PHS(20–90)

**Figure 8:**
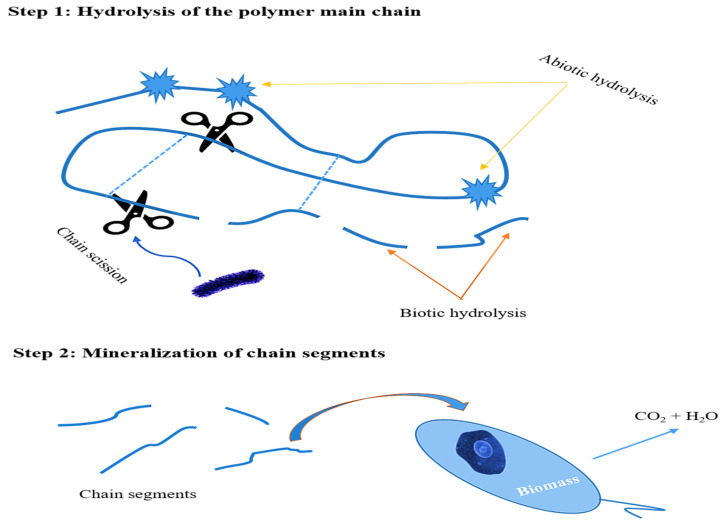
Schematic representation of the expected degradation process for PHS(20–90).

Polymers susceptible to (bio)degradation have attracted a lot of interest over the past decade [53,56]. Enzymatic degradation, for example, could be a method of choice for plastic waste recycling. Within this group of innovative polymers, biopolyesters play a preponderant role due to their hydrolyzable ester bonds. Aromatic polyesters, such as PET, while exhibiting excellent material properties, are very resistant to microbial attack. On the other hand, the PHS(20–90) copolyesters contain sulfur, which is one of the main substrates for “sulfur-eating” bacteria in the soil; thus, they should display appreciable biodegradability by lipases, secreted by microorganisms at neutral pH (7.4), or else under acidic conditions (pH = 2). The rate at which a polymer degrades depends on several factors, including its chemical structure, molar mass, and degree of crystallinity, among other factors. The PHS copolyesters are large molecules including both crystalline and amorphous regions, the latter providing flexibility to the polymers. Degradation should occur in two stages, as shown schematically in Figure 8. In the first step, the polymer chains are cleaved randomly by hydrolase enzymes or abiotic hydrolysis, and the macromolecular chains are cleaved to shorter chain segments more easily attackable by the enzymes. The second step leads to depolymerization, and ultimately to mineralization, namely the transformation of the cleaved chain segments into carbon dioxide and water [69]. 

The thiophene–aromatic copolyesters were explored as candidates, potentially combining good mechanical properties with biodegradability. Another relevant behavior of the PHS(20–90) copolyesters may be the improvement in their hydrolytic degradability, which is expected due to the presence of HDO and DMTD units, even without the participation of enzymes. Indeed, it has been demonstrated that the high resistance to hydrolysis of conventional aromatic polyesters was reduced when thiophene or furan-based diacids were incorporated as modifiers in the chains. To assess the influence of the DMTD content on degradability, the hydrolytic and enzymatic degradation of PHS(20–90) copolyesters was assessed under acidic conditions and in the presence of enzymes at neutral pH, respectively.

**Figure 9:**
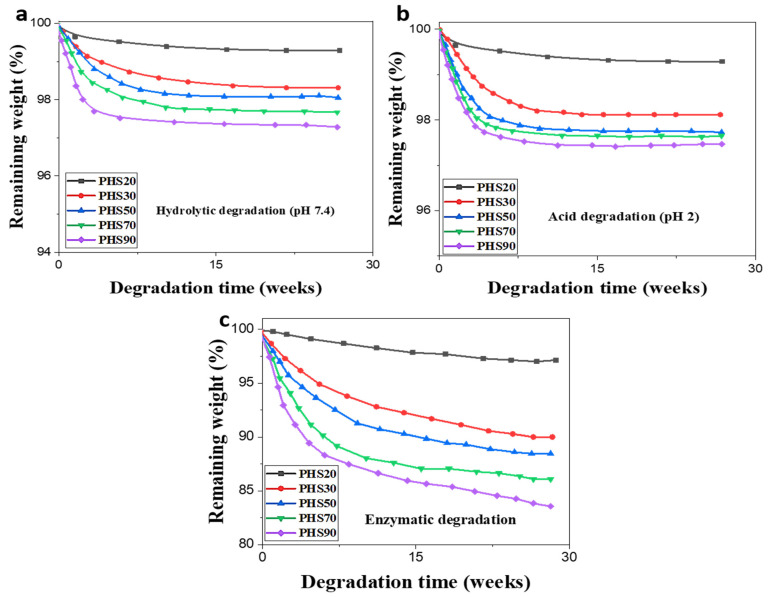
Residual weight versus. incubation time for PHS(20–90) under different conditions: (**a**) pH = 7.4, (**b**) pH = 2, (**c**) enzymatic degradation with Pseudomonas fluorescens lipases (at neutral pH).

The decreases in sample weight during the degradation of PHS(20–90) copolyesters as a function of their incubation time in a different pH and in the presence of lipases are compared in Figure 9a–c. As expected, DMDMT-rich PHS20 was only slightly affected under simple hydrolysis conditions (pH = 2 and pH = 7.4), even after 28 weeks of incubation, and this behavior was barely altered in the presence of lipases at neutral pH. Conversely, PHS(30–90) displayed increasing weight losses as their DMTD content increased under all conditions, but most notably in the presence of lipases, indicating the onset of a slow degradation process (Figure 9). Sample PHS90 displayed the highest weight loss, and SEC monitoring analysis showed the change in molar mass of the sample to decrease from an initial 27,500 g/mol to about 23,100 g/mol after 28 weeks of degradation. Moreover, there were little changes in the molar masses of all the samples after the incubation period. However, a slight increase in dispersity Đ from the initial 2.50 to 3.84 after 28 weeks of sample incubation was observed due to the increase in the low mass fraction, proving the existence of chain-scission occurring in the polyester backbone (Figure 8). Molar mass, M_n_, in the sample dropped from the initial 12,300 g/mol to 7900 g/mol after the incubation period. This observed phenomenon was explained by Koitabashi et al. [70] and Piotr et al. [57], who both noticed that decomposition occurs near the end of the chain in the presence of selective microorganisms, where there are usually more free enzymes attacking the ester groups. Substantial weight loss with almost no change in weight-average molar masses and slight or no change in copolymer composition were observed, indicating the retention of aromatic chain fragments, which may lead to enrichment of low molar mass polymer fraction in the non-degraded aromatic structures induced by DMDMT. It results in sample M_n_ decrease and widening of the molar mass distribution [57]. Additionally, the C–S bonds derived from DMTD units is an added advantage for degradation, since sulfur serves as substrate for microorganisms (sulfur-loving bacteria), thereby promoting hydrolytic degradation. Faster degradation occurred for the samples containing larger amounts of DMTD, in the order PHS90 > PHS70 > PHS50 > PHS30 > PHS20.

It is noteworthy that the degradability of the copolyesters increased significantly in the presence of enzymes under neutral conditions (Figure 9c). The P. fluorescens lipases were tested at neutral pH and 25 °C for optimal hydrolytic activity, and maximum degradation was observed for PHS90, which lost approximately 16% of its initial weight after 28 weeks of incubation, while slower degradation was observed from 0–23 weeks. The conditions were optimized in the experiments to favor the enzymatic degradation, but in a natural environment, these conditions would be difficult to achieve. At longer incubation times (from 23–25 weeks), the enzymatic degradation rate increased rapidly due to the ability of enzymes to assimilate and convert shorter chain segments to waste products such as monomers, CO_2_ and H_2_O.

**Figure 10:**
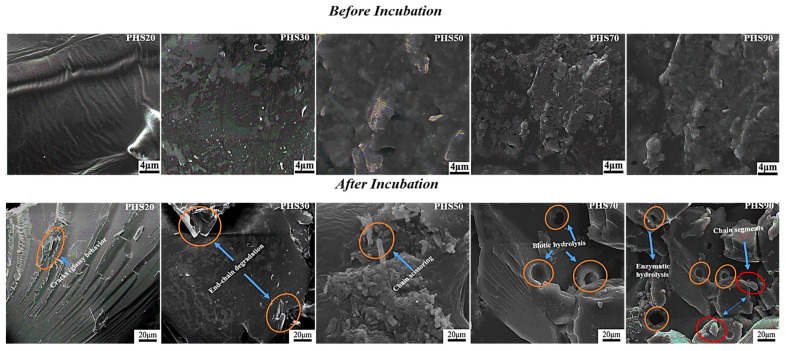
SEM images for PHS(20–90) films before and after 28 weeks of incubation in the enzymatic solution at neutral pH.

The effect of enzymatic degradation was also evaluated at the microscopic level using scanning electron microscopy (SEM) imaging (Figure 10). In the case of PHS20, the recovered material did not display significant changes due to inaccessibility of the ester groups to enzymes, attributed to its high DMDMT content. PHS30 was somewhat more fragile over the incubation period, possibly due to the destruction of chain regularity by the increased amount of DMTD rings. Despite the modest weight losses observed for PHS20 and PHS30, the film surfaces displayed multiple points of attack by enzymes. The PHS50, PHS70 and PHS90 samples displayed further degradability due to their higher DMTD contents. Visible degradation points extending over the whole surface can be observed for these copolyesters, consistent with the significant weight losses observed under optimal conditions (Figure 10). Enzymatic degradation therefore proved to be an efficiency biodegradation method, potentially allowing the monomers to be recovered and reused for the synthesis of polyesters rather than discarded in the environment.

## 3. Experimental Section

### 3.1. Materials

All the procedures using materials sensitive to air or water were carried out under nitrogen atmosphere with standard Schlenk techniques.

Dimethyl 2,5-thiophenedicarboxylate (DMTD, purity > 98% ) was synthesized as described by Wang et al. [46]. The synthesis of dimethyl 2,5-dimethoxyterephthalate (DMDMT, purity > 98%) was described in our previous work [47]. Methanol, ethanol, dimethylformamide, chloroform, and Pseudomonas fluorescens enzymatic solution with an activity above 50 U/mL were purchased from the Wuhan Squire Test Agent Liability Co. All the chemicals were used as received without further purification.

### 3.2. Synthesis of Copolyesters PHS(20–90)

The copolyesters PHS(20–90) (Figure 2) were prepared in a three-neck round-bottom flask equipped with a stirrer, a thermometer, a nitrogen gas inlet, and a condenser. A two-step polycondensation reaction with DMTD, DMDMT, and HDO was applied with a diol to diesters molar ratio of 1.3:1, as provided in Table 1, and 0.15% (based on the diesters mole ratios) antimony trioxide (Sb_2_O_3_) as catalyst. The final products were hard and light-yellow fiber-forming polymers. The polyesters were dissolved in chloroform, precipitated in methanol, and recovered by centrifugation. The products were rinsed by dispersion in 200 mL of water–ethanol (1:1 *v*/*v*), isolated by filtration, and dried for 4 h at 60 °C in a vacuum oven.

### 3.3. Characterization Techniques

(i) FTIR: Fourier transform infrared (FTIR) spectra of the obtained copolyesters PHS(20–90) were obtained from KBr/sample pellets, with a Bruker Vertex 70 spectrometer. The spectra were recorded from 400–4000 cm^−1^, averaging 5 spectra.

(ii) ^1^H and ^13^C NMR: An AVANCE DMX600 spectrometer was used to analyze the composition of the copolyesters with 80 mg/mL solutions of the copolymers in CDCl_3_, using tetramethylsilane (TMS) as internal standard. ^13^C NMR spectra were obtained on a Bruker AM-300 spectrometer at 25.1 MHz and room temperature.

(iii) GPC analysis: A Viscotek TDAmax instrument calibrated with poly(methyl methacrylate) (PMMA) standards was used to obtain the apparent (PMMA-equivalent) polymer number-average molar mass (M_n_), weight-average molar mass (M_w_), and dispersity (Đ = M_w_/M_n_) of the samples, using either tetrahydrofuran (THF) at 25 °C or dimethylformamide (DMF) at 40 °C as eluents, at a flow rate of 1.0 mL/min. Note: the average molar masses (MM) of polyesters calculated using this calibration are slightly estimated with a factor less than two.

(iv) Differential scanning calorimetry (DSC) and thermogravimetric analysis (TGA) measurements: Thermal analysis was carried out on a Netzsch STA 449 F3 Mettler instrument. For the DSC measurements, the sample with a weight of approximately 15 mg was purged with N_2_ at a rate of 50 mL/min. To erase their thermal history, the samples were heated from 25 to 250 °C at 10 °C/min and held for 3 min. They were then quenched to room temperature and heated to 250 °C for a second time. For the TGA measurements, a sample with a weight of approximately 6 mg and a N_2_ flow rate 40 mL/min were used. The samples were heated from 25 to 700 °C at a rate of 10 °C/min.

(v) Tensile testing: The tensile properties were studied on an Instron-1121 tester. The crosshead speed was 5 mm/min, with dumbbell-shaped samples prepared by injection molding. The dimensions of the middle segment were 1.6 mm thickness × 5.0 mm width. For accuracy, a minimum of 5 specimens were tested for each sample; the Young’s modulus, tensile strength, and elongation at break were determined from the average, and the error on the measurements from the standard deviation.

(vi) Degradation: For both hydrolytic and enzymatic degradation studies, films of the copolyesters with a thickness of about 200 µm were prepared by casting from chloroform solutions (0.92 mg/mL). The films were cut into 10 mm diameter disks and dried under a vacuum to constant weight between 20 and 30 mg. The disks were immersed individually in vials at 25 °C containing 10 mL of either sodium phosphate buffer (pH = 7.4, neutral) and 10 mg of lipases from Pseudomonas fluorescens (enzymatic degradation), or in hydrochloric acid buffer at pH 2.0 (hydrolytic degradation). The buffered enzyme solution, with an activity of 50 U/g, was replaced every 72 h to maintain a relatively constant enzymatic activity. At different times over the scheduled incubation period (up to 28 weeks), the disks were withdrawn from the incubation medium, and dried to constant weight to determine mass losses. The effect of incubation with lipases was also evaluated at the microscopic level, by imaging the film surfaces with a Hitachi S-3400N scanning electron microscope (SEM).

## 4. Conclusions

Heterocyclic dimethyl 2,5-thiophenedicarboxylate (DMTD), and aromatic dimethyl 2,5-dimethoxyterephthalate (DMDMT) were successfully used as co-monomers with 1,6-hexanediol (HDO) for the synthesis of copolyesters PHS(20–90) by melt polycondensation. The new thiophene–aromatic copolyesters had satisfactory molecular weights regardless of their composition. All the copolyesters were semi-crystalline, but their glass transition temperature and melting point reached a maximum at an equimolar ratio of DMDMT and DMTD. The copolyesters had melting temperatures ranging from 155 to 183 °C, and the samples displayed good thermal stability up to at least 270 °C. The novelty of these bio-based copolyesters lies in their properties, making them suitable as replacement materials for petroleum-based thermoplastics. Another interesting feature is their high susceptibility to biodegradation at neutral pH in the presence of lipases, with weight losses reaching up to 16%. This biodegradability is of paramount importance for potential applications in domains where self-degradation of the materials is required at the end of their life cycle. The fact that these polymeric materials are susceptible to enzymatic degradation is promising in terms of depolymerization of polymer waste, recycling, to convert waste plastics into higher value bio-products, such as monomers, or ultimately into minerals (CO_2_ and H_2_O).

## Figures and Tables

**Table 1:** Synthesis conditions for copolyesters PHS(20–90).

	Sample				
	PHS20	PHS30	PHS50	PHS70	PHS90
DMTD:DMDMT molar ratio	0.2:0.8	0.3:0.7	0.5:0.5	0.7 0.3	0.9:0.1
Yield (%)	92	94	95	91	93
1st Step (°C)	160	160	170	180	180
Clearing point (h)	3	2.5	2	1.5	1
M_n_ (g/mol)	15,000	18,500	15,500	14,500	12,300
M_w_ (g/mol)	30,100	35,700	38,800	29,500	27,500
Polydispersity index (Đ)	2.00	2.01	2.50	2.11	2.31
H_a_ (mol %)	35.3	44.3	50.8	59.4	63.1
H_b_ (mol %)	64.7	55.7	49.2	40.6	36.9

_a_ mole composition of 2,5-thiophenedicarboxylate (DMDT) in synthesized copolyesters determined by using ^1^H NMR spectroscopy (see below the corresponding ^1^H NMR spectra). _b_ mole composition of 2,5-dimethoxyterephthalate (DMDMT) in synthesized copolyesters determined by using ^1^H NMR spectroscopy. **1st Step:** Esterification temperature (°C). **2nd Step:** Polymerization temperature for the copolyesters 230 °C.

**Table 2:** Characteristic FTIR stretching signals in the copolymers.

Wavenumber [cm^−1^].	Characteristic Group
3120	thiophene
2927, 2855	-CH_2_- from aliphatic chains
1720	-C(O)- from ester groups
1270	-C-(O)-O-C- from ester groups (sp^2^)
1078	-C-C-O- from ester groups (sp^3^)

**Table 3:** Characteristic ^1^H NMR signals for copolyesters (see Figure 4 for reference to Label letters).

Label	Chemical Shift [ppm]	Group
a	7.77–7.80	TH-H
b	7.55–7.62	Ar-H
c	3.81–3.88	-O-CH_3_
d, e	4.30–4.43	TH-O-CH_2_-CH_2_-O-Ar
f	1.79	-O-CH_2_-CH_2_
g	1.45	-O-CH_2_-CH_2_-CH_2_-

**TH** = Thiophene ring; **Ar** = Phenyl ring.

**Table 4:** Thermal analysis results for copolyesters PHS(20–90).

Copolyesters	T_d, 5%_ [°C]	T_d, 50%_ [°C]	T_d, max_ [°C]	T_m_ [°C]	T_g_ [°C]	∆H_c_ [J/g]	R_700_ [wt %]
PHS20	284	395	410	175	85	−55.6	6.9
PHS30	319	409	412	166	89	−56.4	5.8
PHS50	331	419	426	183	100	−56.8	3.5
PHS70	280	392	406	163	82	−51.2	5.7
PHS90	277	387	395	155	70	−50.4	4.7

**Table 5:** Comparison of the tensile properties of copolyesters PHS(20–90) and commercial polymers.

Sample	Tensile Modulus [MPa]	Tensile Strength [MPa]	Elongation at Break [%]
PET [45,47]	1137	60	5.04
HDPE [45]	1670 ± 54	52.70 ± 3.7	188 ± 19
LDPE [46]	364 ± 4	14.9 ± 2	69 ± 4
PHS20	1185 ± 12	37 ± 2	327 ± 9
PHS30	1273 ± 12	42 ± 2	335± 9
PHS50	1552 ± 12	80 ± 2	370 ± 9
PHS70	895 ± 12	27 ± 2	298 ± 9
PHS90	836 ± 12	23 ± 2	282 ± 9

## Data Availability

Not applicable.

## Sample Availability

Samples of the different compounds are available from the authors.
